# Nano-Curcumin Prevents Copper Reproductive Toxicity by Attenuating Oxidative Stress and Inflammation and Improving Nrf2/HO-1 Signaling and Pituitary-Gonadal Axis in Male Rats

**DOI:** 10.3390/toxics10070356

**Published:** 2022-06-30

**Authors:** Wedad S. Sarawi, Ahlam M. Alhusaini, Laila M. Fadda, Hatun A. Alomar, Awatif B. Albaker, Hanan K. Alghibiwi, Amjad S. Aljrboa, Areej M. Alotaibi, Iman H. Hasan, Ayman M. Mahmoud

**Affiliations:** 1 Pharmacology and Toxicology Department, Faculty of Pharmacy, King Saud University, Riyadh 11451, Saudi Arabia; aelhusaini@ksu.edu.sa (A.M.A.); lfadda@ksu.edu.sa (L.M.F.); hetalomar@ksu.edu.sa (H.A.A.); abaker@ksu.edu.sa (A.B.A.); halghibiwi@ksu.edu.sa (H.K.A.); 441203053@student.ksu.edu.sa (A.S.A.); 442204129@student.ksu.edu.sa (A.M.A.); ihasan@ksu.edu.sa (I.H.H.); 2 Department of Pharmacology and Toxicology, College of Pharmacy, Umm Al-Qura University, Mecca 21955, Saudi Arabia; 3 Physiology Division, Zoology Department, Faculty of Science, Beni-Suef University, Beni-Suef 62514, Egypt; 4 Department of Life Sciences, Faculty of Science & Engineering, Manchester Metropolitan University, Manchester M1 5GD, UK

**Keywords:** curcumin, reproductive toxicity, copper sulfate, steroidogenesis, oxidative stress

## Abstract

Copper is essential for several cellular processes and is an important catalytic factor for many proteins. However, excess copper can provoke oxidative stress and reproductive toxicity. This study evaluated the effect of liposomal nano-curcumin (N-CUR) and CUR on testicular oxidative injury, inflammation, and apoptosis, and altered steroidogenesis and Nrf2/HO-1 signaling induced by copper sulfate (CuSO4). Rats received CuSO4 and N-CUR or CUR via oral gavage for 7 days. CuSO4 induced histopathological changes and altered pituitary-gonadal axis manifested by decreased serum gonadotropins and testosterone. Testicular steroidogenesis genes (StAR, 3β-HSD, CYP17A1, and 17β-HSD) and androgen receptor (AR) were downregulated in rats that received CuSO4. N-CUR and CUR prevented testicular tissue injury, increased circulating FSH, LH, and testosterone, and upregulated testicular steroidogenesis genes and AR. Additionally, N-CUR and CUR decreased testicular MDA, NO, NF-κB, iNOS, TNF-α, Bax, and caspase-3 while enhanced Bcl-2, Nrf2, and the antioxidants GSH, HO-1, SOD, and catalase. In conclusion, N-CUR and CUR prevented CuSO4-induced reproductive toxicity in male rats by suppressing oxidative injury and inflammatory response and boosting steroidogenesis, sex hormones, and Nrf2/HO-1 signaling. N-CUR was more effective in ameliorating tissue injury, oxidative stress, inflammation, and apoptosis and enhancing steroidogenesis and Nrf2/HO-1 than the native form.

## 1. Introduction

Copper (Cu) is an essential element for maintaining several physiological functions such as energy metabolism, protein homeostasis, blood clotting, and the synthesis of neurotransmitters [1,2]. The ion of this redox-active metal is essential for many proteins and enzymes, including ceruloplasmin and superoxide dismutase (SOD), and has a key role in the development of the reproductive system, skin, hair, and bone [3]. The body Cu is maintained within normal levels through the regulation of its absorption and excretion via precise regulatory mechanisms [4]. Cu as a metal is widely used in several industries, including electronics, building materials, wood preservatives, and intrauterine contraceptive devices [5,6]. Owing to its widespread use, exposure to Cu can occur frequently and lead to toxic effects upon exceeding physiological levels. Cu could be absorbed through the skin, lungs, and intestine resulting in toxic effects in different organs, including the liver, kidney, and nervous system [7,8]. Hepatotoxicity, rhabdomyolysis, arrhythmia, seizures, kidney injury, and neurological disorders are excess Cu-linked clinical manifestations [9,10]. Cu was detected in several tissues of aquatic animals and hence has been graded as an environmental pollutant [11,12]. Copper sulfate (CuSO_4_) is one of the Cu compounds that possess fungicidal and bactericidal properties. CuSO_4_ is used to minimize the contamination risk in tissue culture and is employed in agriculture as a pesticide to repel pests and improve crop yield. However, its intentional or accidental intoxication could result in multiorgan toxicity that might be fatal [13]. Accordingly, exposure of rats to CuSO_4_ resulted in increased Cu levels in the liver and kidney and subsequently impairment and damage of these organs [14]. Recent work from our lab has demonstrated neurotoxicity and cardiac injury in rats exposed to CuSO_4_ [15,16].

Reproductive toxicity is one of the manifestations of Cu as reported in several in vivo and in vitro experiments [17,18,19,20]. The effects of Cu on male fertility include significant decrease in the number, motility, and viability of spermatozoa [21]. The intake of Cu, even at low doses, induced adverse effects on the morphology of testis in male mice [22]. Reduced levels of circulating testosterone, follicle-stimulating hormone (FSH), and luteinizing hormone (LH) have been demonstrated in immature male rats exposed to Cu for 26 days [23]. Although the effects of Cu on male fertility are not fully understood, excessive reactive oxygen species (ROS) and oxidative stress could play a significant role [19,24]. Given its rich content of highly unsaturated fatty acids, the testis is vulnerable to oxidative injury [25]. Accordingly, studies have demonstrated that Cu upregulates genes of the oxidative phosphorylation pathway and the generation of ROS and decreases antioxidant defenses and spermatogenesis [17,18]. Therefore, mitigating oxidative stress might attenuate Cu-induced testicular injury.

Curcumin (CUR), the principal curcuminoid of turmeric, is a non-polar polyphenol with potent antioxidant and anti-inflammatory properties [26,27]. CUR is used in dietary supplements, cosmetics, flavored beverages and other applications [28]. It has been virtually studied for several diseases and showed antimicrobial [29], anti-diabetic [30], nephroprotective [31], hepatoprotective [32,33,34,35,36], and antitumor efficacies [37]. It is a bis-α,β-unsaturated β-diketone with aromatic rings functionalized with methoxy and hydroxy groups. The presence of conjugated double bonds in CUR permits effective electron donation and counteracting ROS formation [38]. Therefore, CUR can effectively suppress ROS generation and prevent oxidative injury. CUR has been evidenced to act in a multi-targeted fashion through its ability to target bioactive proteins in various signaling pathways or its epigenetic modulating power [39,40]. The protective efficacy of CUR against testicular injury induced by titanium dioxide [41], lead acetate [42], and doxorubicin (DOX) [43] has been reported; however, its protective efficacy against testicular injury caused by CuSO_4_ has not been investigated yet. In addition, the rapid metabolism and poor systemic bioavailability of CUR are the main drawbacks that can limit its therapeutic applications [44]. Nanoformulations greatly improved the properties of CUR by amending its cellular uptake, permeability, and plasma bioavailability [45]. Liposomal CUR nanoformulation exhibited a better bioavailability in different diseases, highlighting that liposomes are optimal delivery vehicles [46]. Liposomes are spherical vesicles constituted by phospholipid bilayers where the polar groups are oriented to the outer and inner aqueous phases. Liposomes have a high propensity to carry both hydrophobic and hydrophilic drugs and improve their accumulation in tissues and therapeutic actions and decrease side effects [46,47]. This study explored the efficacy of liposomal nano-CUR (N-CUR) against oxidative stress, inflammation, and testicular injury induced by CuSO_4_, pointing to the involvement of nuclear factor erythroid 2-related factor 2 (Nrf2)/heme oxygenase-1 (HO-1) signaling. Nrf2 is a cytoprotective factor that controls the transcription of antioxidant defenses and prevents oxidative damage and inflammation [48].

## 2. Materials and Methods

### 2.1. Chemicals and Reagents

Liposomal N-CUR and deferoxamine (DFO) were supplied by Lipolife (Essex, UK) and Novartis Pharma AG (Rotkreuz, Switzerland), respectively. CUR, CuSO_4_, thiobarbituric acid, sodium dodecyl sulfate (SDS), primers, Griess reagent, pyrogallol, agarose, reduced glutathione (GSH), hematoxylin and eosin (H&E), bisacrylamide, hydrogen peroxide (H_2_O_2_) and carboxymethylcellulose (CMC) were purchased from Sigma (St. Louis, MO, USA). Protein assay kit was obtained from BioBasic (Markham, Canada), tumor necrosis factor (TNF)-α ELISA kit was supplied by R&D Systems (Minneapolis, MN, USA), and testosterone ELISA kit was purchased from Cusabio (Wuhan, China). FSH and LH ELISA kits and antibodies against Nrf2, HO-1, androgen receptor (AR), and β-actin were provided by Novus Biologicals (Centennial, CO, USA). Other chemicals were obtained from standard suppliers.

### 2.2. Experimental Animals and Treatments

Forty male Wistar rats (180–200 g) were supplied by the Animals Care Centre (King Saud University). The rats were housed in standard cages under a 12-h light/dark cycle and standard temperature and humidity. The animals had free access to food and water and were allocated into 5 groups (*n* = 8) after acclimatization for one week. Rats in group I served as control and received 1% CMC. Rats in groups II-V received 100 mg/kg CuSO_4_ [14], and groups III, IV and V were supplemented with 23 mg/kg DFO [49], 80 mg/kg CUR [13,49], and 80 mg/kg N-CUR [13,49], respectively. All treatments were suspended in 1% CMC and given orally for 7 days. On day 8, the rats were anesthetized wiht ketamine/xylazine, and blood was collected via cardiac puncture for serum preparation. The rats were sacrificed and the testes were removed and washed in cold phosphate-buffered saline (PBS). Pieces from the testes were fixed in Bouin’s solution while other samples were homogenized (10% *w*/*v*) in Tris-HCl buffer (pH 7.4). The homogenate was centrifuged, and the supernatant was collected for the determination of malondialdehyde (MDA), nitric oxide (NO), GSH, SOD, catalase (CAT), and TNF-α. Other samples were kept frozen at −80 °C for RNA isolation and western blotting.

### 2.3. Assay of Testosterone, Gonadotropins, and TNF-α

Testosterone, FSH, and LH were assayed in the serum and TNF-α was determined in the testicular tissue supernatant using specific ELISA kits according to instructions of the suppliers.

### 2.4. Assay of MDA, NO and Antioxidants

MDA and NO were assayed in the testicular tissue supernatant following the methods of Ohkawa et al. [50] and Grisham et al. [51], respectively. GSH was determined according to the method of Ellman [52], and the activities of SOD and CAT were determined according to the methods described by Marklund and Marklund [53] and Cohen et al. [54], respectively.

### 2.5. Histopathological Examination

The testes samples were fixed in Bouin’s solution for 24 h, and then processed for paraffin wax embedding. Five-μm sections were cut using a rotary microtome. The sections were stained with H&E for microscopic examination.

### 2.6. Gene Expression

Changes in the expression of nuclear factor-kappaB (NF-κB) p65, inducible NO synthase (iNOS), B cell lymphoma-2 (BCL-2), BCL-2-associated X protecin (BAX), caspase-3, and steroidogenic acute regulatory protein (StAR), 3β-Hydroxysteroid dehydrogenase (HSD), 17β-HSD, and cytochrome P450 17A1 (CYP17A1) were evaluated using qRT-PCR [55]. TRIzol (ThermoFisher Scientific, Waltham, MA, USA) was used to isolate RNA which was treated with RNase-free DNase (Qiagen, Hilden, Germany) and quantified on a nanodrop. RNA samples with A260/A280 nm ratio of ≥1.8 were selected for cDNA synthesis which was amplified using Maxima SYBR Green/ROX qPCR master mix (ThermoFisher Scientific, Waltham, MA, USA) and the primers in Table 1. The obtained data were analyzed using the 2^−ΔΔCt^ method [56] and normalized to β-actin.

### 2.7. Western Blotting

To evaluate changes in the expression of Nrf2, HO-1, and proliferating cell nuclear antigen (PCNA), the tissue was homogenized in RIPA buffer, and the homogenate was centrifuged at 10,000 rpm. The concentration of protein was assayed in the supernatant using a Bradford protein assay kit. Fifty µg protein was subjected to 10% SDS/PAGE and the separated protein bands were transferred to nitrocellulose membranes. After blocking in 5% milk in tris-buffered saline tween (TBST), the membranes were incubated overnight at 4 °C with anti-Nrf2, anti-HO-1, anti-AR, or anti-β-actin. The membranes were washed three times in TBST and probed with the secondary antibodies for 1 h at room temperature. After washing with TBST, the bands were developed with Clarity™ Western ECL Substrate from BIO-RAD (Hercules, CA, USA) and visualized using ImageQuant LAS 4000. The band intensity was quantified using ImageJ (version 1.32j, NIH, Bethesda, MD, USA).

### 2.8. Statistical Analysis

The results are expressed as mean ± standard error of the mean (SEM). Statistical analysis and multiple comparisons were performed by one-way ANOVA and Tukey’s post-hoc test using GraphPad Prism 8. A *p* value <0.05 was considered significant.

## 3. Results

### 3.1. N-CUR and CUR Enhance the Pituitary-Gonadal Axis and Prevent Testicular Injury in CuSO_4_-Administered Rats

**Figure 1:**
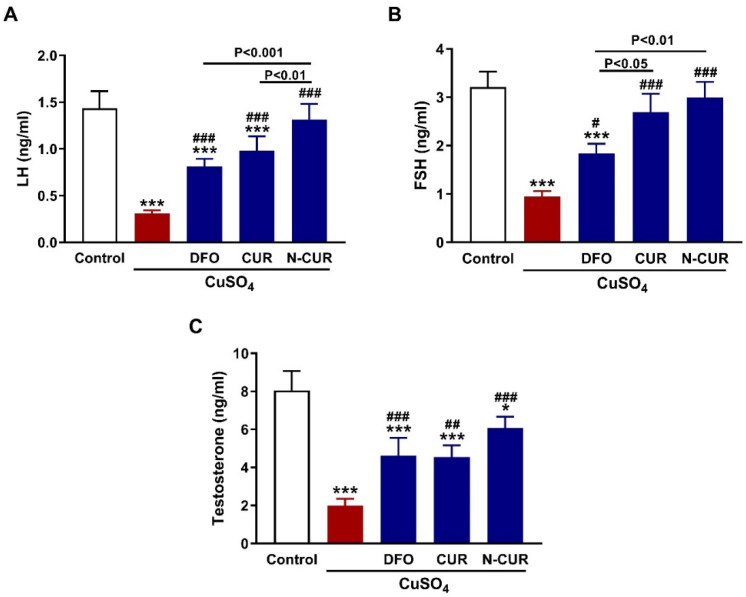
N-CUR and CUR enhance the pituitary-gonadal axis in Cu-intoxicated rats. CUR, N-CUR, and DFO increased serum (**A**) LH, (**B**) FSH, and (**C**) testosterone in rats that received CuSO_4_. Data are mean ± SEM, (*n* = 8). * *p* < 0.05 and *** *p* < 0.001 versus Control. ^#^ *p* < 0.05, ^##^ *p* < 0.01, and ^###^ *p* < 0.001 versus CuSO_4_.

The circulating levels of LH, FSH, and testosterone were assayed to evaluate the impact of CuSO_4_ on the pituitary-gonadal axis and the protective effect of N-CUR and CUR (Figure 1). CuSO_4_-treated rats exhibited a decrease in LH (Figure 1A), FSH (Figure 1B), and testosterone (Figure 1C) as compared to the control (*p* < 0.001). DFO, CUR, or N-CUR alleviated serum LH, FSH, and testosterone in CuSO_4_-administered rats. CUR increased serum FSH significantly (*p* < 0.05) when compared to DFO, while N-CUR alleviated both LH and FSH levels.

**Figure 2:**
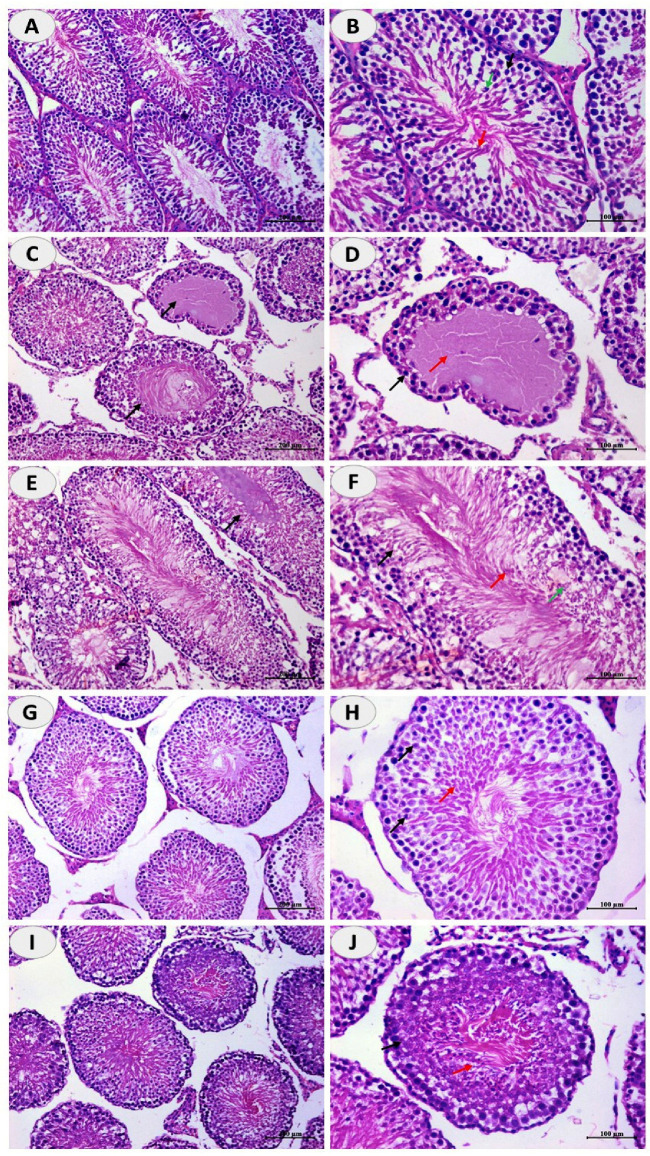
N-CUR and CUR prevent Cu-induced testicular tissue injury. Photomicrographs of H&E-stained sections in the testis of (**A**,**B**) control rats showing normal histological architecture, (**C**,**D**) Cu-intoxicated rats showing degenerative changes and loss of spermatogenic series, and spermatozoa, and (**E**–**J**) Cu-intoxicated rats treated with DFO (**E**,**F**), CUR (**G**,**H**), and N-CUR (**I**,**J**) showing normal seminiferous tubules with spermatogonia, primary spermatocytes, spermatids, and spermatozoa. (×200: **A**,**C**,**G**,**E**,**I**. ×400: **B**,**D**,**F**,**H**,**J**).

Examination of the testis of control rats revealed normal seminiferous tubules with normal spermatogonia, spermatocytes, spermatids and a large number of spermatozoa (Figure 2A,B). Oral supplementation of CuSO_4_ induced degeneration of the seminiferous tubules and loss of spermatogenic series and complete lack of mature spermatozoa as shown in Figure 2C,D. Concurrent treatment of the rats with DFO (Figure 2E,F), CUR (Figure 2G,H), or N-CUR (Figure 2I,J) prevented CuSO_4_-induced testicular tissue injury and the seminiferous tubules appeared to possess the normal histological architecture with spermatogonia, primary spermatocytes, spermatids, and spermatozoa.

### 3.2. N-CUR and CUR Upregulate Steroidogenesis and AR in CuSO_4_-Administered Rats

**Figure 3:**
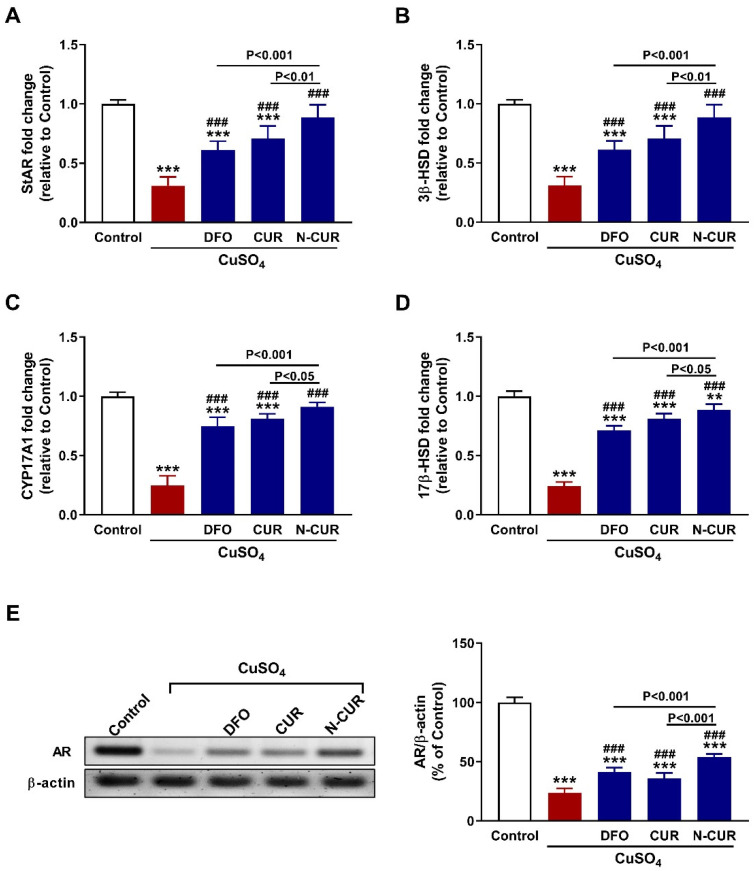
N-CUR and CUR upregulate steroidogenesis and AR in Cu-intoxicated rats. Treatment with DFO, CUR, and N-CUR increased (**A**) StAR, (**B**) 3β-HSD, (**C**) CYP17A1, and (**D**) 17β-HSD mRNA abundance, and (**E**) AR protein expression. Data are mean ± SEM, (*n* = 8). *** *p* < 0.001 versus Control and ^###^ *p* < 0.001 versus CuSO_4_.

The effect of CuSO_4_ on steroidogenesis and the ameliorative potential of N-CUR and CUR were evaluated by determining the mRNA abundance of StAR (Figure 3A), 3β-HSD (Figure 3B), CYP17A1 (Figure 3C), and 17β-HSD (Figure 3D), and the protein expression of AR (Figure 3E). CuSO_4_ downregulated the mRNA abundance of StAR, 3β-HSD, CYP17A1, and 17β-HSD, as well as AR protein in the testis of rats (*p* < 0.001). Both forms of CUR as well as DFO upregulated the expression of steroidogenesis genes and AR (*p* < 0.001). N-CUR upregulated StAR, 3β-HSD, CYP17A1, 17β-HSD, and AR significantly when compared to either DFO or CUR (Figure 3).

### 3.3. N-CUR and CUR Attenuate Testicular Oxidative Stress in CuSO_4_-Administered Rats

**Figure 4:**
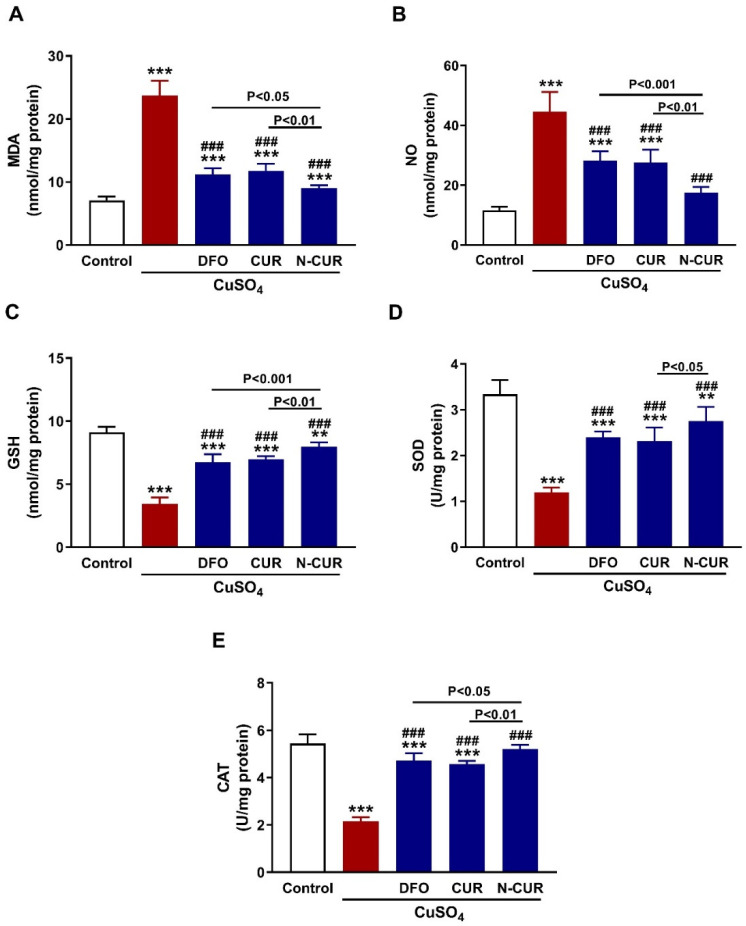
N-CUR and CUR attenuate testicular oxidative stress in Cu-intoxicated rats. Treatment with DFO, CUR, and N-CUR decreased (**A**) MDA and (**B**) NO and increased (**C**) GSH, (**D**) SOD, and (**E**) CAT. Data are mean ± SEM, (*n* = 8). ** *p* < 0.01 and *** *p* < 0.001 versus Control, and ^###^ *p* < 0.001 versus CuSO_4_.

Both MDA (Figure 4A) and NO (Figure 4B) levels showed a significant elevation in the testis of CuSO_4_-administered rats (*p* < 0.001). In contrast, testicular GSH (Figure 4C), SOD (Figure 4D), and CAT (Figure 4E) were significantly decreased following the administration of CuSO_4_ (*p* < 0.001). All treatments markedly decreased testicular MDA and NO, and increased GSH, SOD and CAT (*p* < 0.001). N-CUR was more effective in ameliorating oxidants, GSH and CAT than DFO and CUR, and increasing SOD activity than CUR.

### 3.4. N-CUR and CUR Upregulate Nrf2/HO-1 Signaling in CuSO_4_-Administered Rats

**Figure 5:**
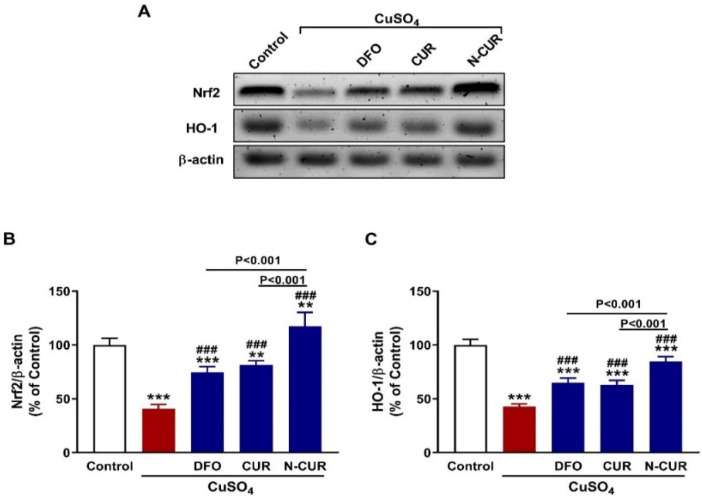
N-CUR and CUR upregulate Nrf2/HO-1 signaling in Cu-intoxicated rats. (**A**–**C**) Treatment with DFO, CUR, and N-CUR increased testicular Nrf2 and HO-1 protein levels. Data are mean ± SEM, (*n* = 8). ** *p* < 0.01 and *** *p* < 0.001 versus Control, and ^###^ *p* < 0.001 versus CuSO_4_.

Nrf2 and HO-1 in the testis of rats were determined using western blotting that revealed significant downregulation of both proteins in CuSO_4_-administered rats (*p* < 0.001; Figure 5A–C). Treatment of these rats with CUR and N-CUR markedly increased the expression of testicular Nrf2 and HO-1. Of note, N-CUR upregulated Nrf2 and HO-1 significantly (*p* < 0.001) when compared with either DFO or CUR.

### 3.5. N-CUR and CUR Mitigate Testicular Inflammation in CuSO_4_-Administered Rats

**Figure 6:**
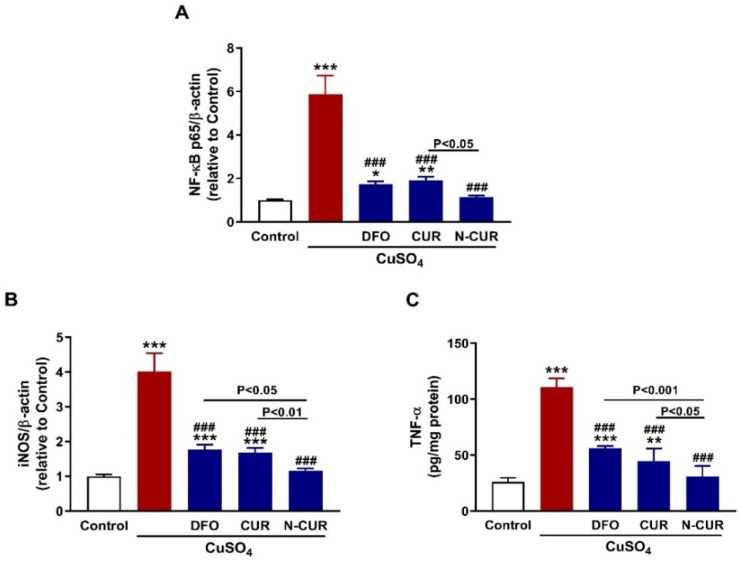
N-CUR and CUR mitigate testicular inflammation in Cu-intoxicated rats. CUR, N-CUR, and DFO decreased (**A**) NF-κB p65 and (**B**) iNOS mRNA abundance, and (**C**) TNF-α protein. Data are mean ± SEM, (*n* = 8). * *p* < 0.05, ** *p* < 0.01 and *** *p* < 0.001 versus Control, and ^###^ *p* < 0.001 versus CuSO_4_.

The inflammatory response provoked in the testis was evaluated through the assessment of NF-κB p65, iNOS, and TNF-α expression as depicted in Figure 6. CuSO_4_-administered rats exhibited a significant upregulation of NF-κB p65 (Figure 6A) and iNOS (Figure 6B) mRNA abundance and TNF-α protein (Figure 6C) (*p* < 0.001). CUR, DFO, and N-CUR prevented the increase in NF-κB p65, iNOS, and TNF-α in the testis of CuSO_4_-administered rats (*p* < 0.001). Notably, N-CUR was more effective in decreasing NF-κB p65 than CUR and iNOS, and TNF-α than DFO and CUR.

### 3.6. N-CUR and CUR Prevent Testicular Apoptosis in CuSO_4_-Administered Rats

**Figure 7:**
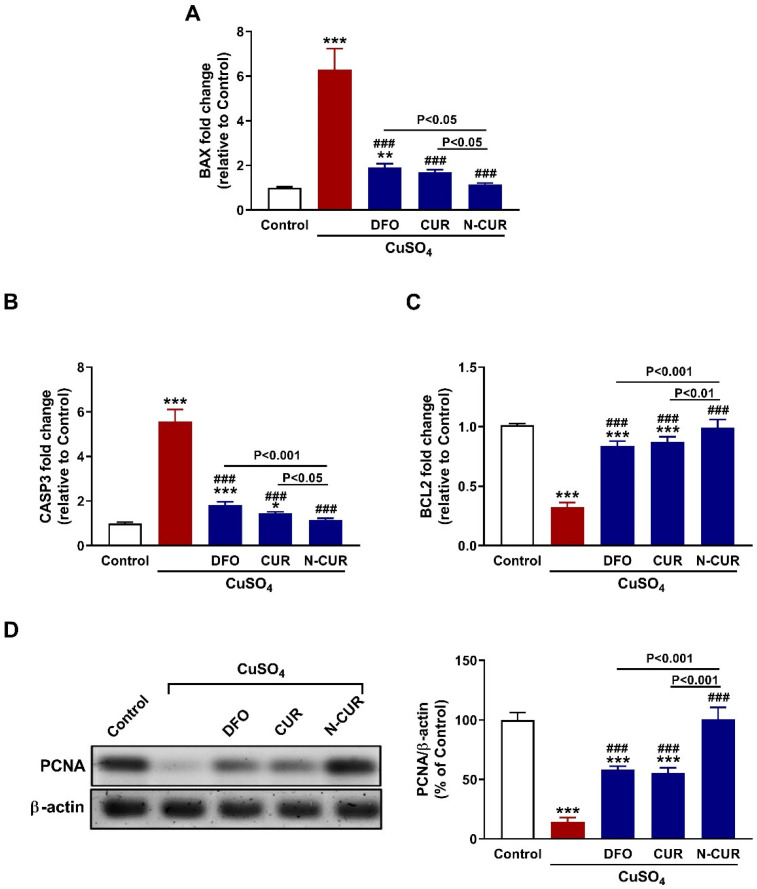
N-CUR and CUR prevent testicular apoptosis in Cu-intoxicated rats. CUR, N-CUR, and DFO downregulated (**A**) Bax and (**B**) caspase-3, and upregulated (**C**) Bcl-2 and (**D**) PCNA in the testis of rats. Data are mean ± SEM, (*n* = 8). * *p* < 0.05, ** *p* < 0.01 and *** *p* < 0.001 versus Control, and ^###^ *p* < 0.001 versus CuSO_4_.

To evaluate the protective effect of N-CUR and CUR against Cu-induced testicular apoptosis, we assayed the mRNA abundance of Bax, caspase-3, and Bcl-2 along with the protein expression levels of PCNA. Administration of CuSO_4_ provoked upregulation of testicular Bax (Figure 7A) and caspase-3 (Figure 7B) and downregulated Bcl2 (Figure 7C) and PCNA (Figure 7D) as compared to the control rats (*p* < 0.001). All treatment agents ameliorated the expression of all assayed markers significantly. In comparison with either CUR or DFO, N-CUR was more effective in ameliorating the expression of apoptosis markers and PCNA.

## 4. Discussion

Copper is an essential cofactor for antioxidant enzymes, immune function and a plethora of vital cellular processes [2]. However, its extensive usage in many enterprises, such as, electronics, pesticides, building and transportation materials, water pipes and others incurred a notable eco-environmental contamination by polluting land and water resources [5,6,57]. Exposure to Cu compounds has been associated with adverse effects, including testicular oxidative stress, and apoptosis, along with declined sperm quality [19,24]. CUR has shown potent antioxidant properties and protected the testis against injury induced by various agents [26,27,41,42,43]. Herein, we evaluated the role of CUR and N-CUR in attenuating CuSO_4_-induced testicular injury in rats, pointing to changes in steroidogenesis, Nrf2/HO-1 signaling, and inflammatory and apoptosis markers.

Exposure to CuSO_4_ caused testicular tissue damage and disrupted hormones of the pituitary-gonadal axis. CuSO_4_ ingestion resulted in degenerative changes, germ cells depletion and significantly reduced the number of spermatozoa in the seminiferous tubules and decreased the circulating levels of gonadotropins and testosterone. Accordingly, recent studies demonstrated declined serum LH and FSH in pigs [24] and testosterone in rats [19] exposed to Cu compounds, including CuSO_4_. FSH and LH are important for the proliferation and normal function of Sertoli cells and Leydig cells, respectively. FSH is necessary for the proliferation, maturation, and function of Sertoli cells which generate signals for the initiation and maintenance of spermatogonia [58]. Therefore, normal secretion of FSH is essential for maintaining spermatogenesis [58]. LH induces the proliferation of Leydig cells and stimulates the secretion of testosterone which maintain the structural and functional integrity of reproductive organs and male accessory glands [58]. The observed decrease in serum testosterone in rats exposed to CuSO_4_ could be directly ascribed to insufficient LH secretion and/or reduced responsiveness of Leydig cells to LH.

Moreover, our findings introduced new information on the effect of Cu exposure on steroidogenesis. Rats that received CuSO_4_ exhibited a significant decrease in testicular StAR, 3ß-HSD, CYP17A1 and 17ß-HSD mRNA abundance. These enzymes are essential for maintaining steroidogenesis and male fertility. StAR controls the rate-limiting step of testosterone biosynthesis by promoting the transport of cholesterol into the mitochondria [59], and its inhibition can stop the steroidogenic machinery in Leydig cells [60]. Within the mitochondria, CYP11A1 converts cholesterol to pregnenolone which is then hydroxylated by CYP17A1. 3ß-HSD and 17ß-HSD catalyze the subsequent steps of testosterone biosynthesis [61]. The reduced levels of FSH, LH, and testosterone along with the downregulated steroidogenic genes confirmed the endocrine and reproductive toxic effect of CuSO_4_.

CUR and N-CUR prevented testicular injury, ameliorated sex hormone levels, and enhanced steroidogenesis in rats that received Cu. In addition, both CUR and N-CUR upregulated the expression of AR in the testis of rats. ARs, ligand-activated nuclear receptors, are highly expressed in the testes, particularly Sertoli cells, and play a central role in maintaining spermatogenesis. Upon binding of testosterone or its metabolite, AR dissociates from heat-shock proteins, dimerizes, and binds to its response element in the nucleus to promote the expression of genes responsible for the maintenance, growth and maturation of male gametes [62]. The total or conditional knockout of AR caused severe defects in reproductive development and spermatogenesis in mice [63]. Although the effect of CuSO_4_ on spermatogenesis has been previously reported [19], we introduced evidence that it downregulates AR in the testis and that CUR and its nanoform can increase testosterone biosynthesis and AR. The ability of CUR to prevent testicular damage caused by various toxicants, including titanium dioxide [41], lead [42], and DOX [43] has been previously demonstrated. In these studies, the protective effect of CUR prevented histopathological alterations, increased testosterone release and enhanced sperm parameters. Here, N-CUR showed a more potent protective effect when compared to the native form, an effect that is directly attributed to the improved properties by nanoformulation. It is noteworthy pointing out that the chelating agent DFO exerted a protective effect against CuSO_4_-induced testicular toxicity. Hence, the chelating property of CUR might be involved in its protective effect against Cu toxicity. In support of this notion, the chelate complex of CUR with Cu(II) was studied by employing quantum chemical computations as reported by Balasubramanian [64]. The primary site for chelating Cu(II) is the β-diketone bridge of CUR and the chelation mechanism involves the loss of an enolic proton of CUR [64].

Oxidative stress has been suggested to play a key role in Cu toxicity [15,16], and some researchers have demonstrated that its testicular toxicity occurs via provoking oxidative damage [19,24]. Cu is able to cycle easily between unstable reduced and stable oxidized states and facilitate redox reactions. However, this renders it toxic because of the surplus release of hydroxyl radicals [65,66]. Also, Cu can upregulate genes of the oxidative phosphorylation pathway and enhance the generation of mitochondrial ROS, decrease antioxidants, and suppress spermatogenesis [17,18]. ROS are potent oxidizers that cause oxidative damage of lipids, DNA and proteins, and provoke cell death [66]. Accordingly, Cu increased testicular MDA and NO and suppressed the antioxidants GSH, SOD and CAT, demonstrating oxidative stress. The well-documented antioxidant and radical-scavenging properties of CUR [32,33,38,67] are involved in its protective effect against CuSO_4_-induced testicular toxicity. The antioxidant potency of CUR was demonstrated in animal models of testicular toxicity induced by lead acetate [42], and DOX [43] where it decreased MDA and enhanced GSH and antioxidant enzymes. In this study, N-CUR decreased MDA and NO, and boosted antioxidant defenses in the testis of CuSO_4_-administered rats significantly as compared to the native form, demonstrating its enhanced radical-scavenging activity.

Besides its radical scavenging properties, activation of Nrf2/HO-1 signaling might be involved in the protective effect of CUR and N-CUR against CuSO_4_-induced testicular toxicity. The results revealed that exposure to Cu downregulated testicular Nrf2 and HO-1, an effect that was prevented in rats supplemented either CUR or N-CUR. Nrf2 is a redox-sensitive factor located in the cytoplasm sequestered by Keap-1. Upon activation by ROS or electrophiles, it dissociates from Keap-1 and translocates into the nucleus where it binds to ARE and stimulate the expression of detoxifying, cytoprotective and antioxidant genes, including HO-1 [48]. Activation of Nrf2/HO-1 signaling protected the testis against heavy metal-induced oxidative stress, inflammation, and apoptosis [55,68]. CUR upregulated Nrf2/HO-1 and prevented oxidative stress in the testis of cadmium-intoxicated mice [68]. The ability of CUR to upregulate Nrf2 signaling has been investigated in different in vitro and in vivo models (reviewed in [69]). In consistence with the results of antioxidant enzymes, N-CUR was more effective in upregulating testicular Nrf2/HO-1 signaling in CuSO_4_-administered rats.

Inflammation and apoptosis are implicated in the toxic effects of Cu, including neurotoxicity [15], cardiotoxicity [16] and testicular toxicity [24]. Here, NF-κB, iNOS, and TNF-α were significantly upregulated in the testes of rats that received CuSO_4_, denoting an inflammatory response. The generation of ROS by Cu is the main culprit behind the developed inflammation. Excess ROS can activate many signaling molecules, including NF-κB which subsequently elicits the transcription of iNOS and inflammatory cytokines [70]. Recently, we have reported the activation of TLR4/NF-κB and MAPK signaling and the release of pro-inflammatory cytokines in the cardiac tissue of Cu-administered rodents [16]. In conjunction with inflammation, testicular apoptosis was observed in CuSO_4_-administered rats. CuSO_4_ increased Bax and caspase-3 and decreased the anti-apoptotic marker Bcl-2. Similar findings have been previously reported in the brain and heart of rats treated with CuSO_4_ [15,16]. ROS and pro-inflammatory mediators can activate Bax which promotes the loss of mitochondrial membrane potential and subsequently cytochrome c is released to the cytosol where it activates the executioner caspase-3 [71]. Caspase-3 provokes DNA fragmentation, degradation of cytoskeletal proteins and further release of cytochrome c, leading to cell death [72]. Furthermore, CuSO_4_ decreased the expression of PCNA in the testes of rats. PCNA is related to DNA synthesis, chromosome recombination and RNA transcription, and is involved in the proliferation and differentiation of spermatogonia [73]. The positive expression of PCNA is conductive to DNA replication, cell proliferation and smooth spermatogenesis [73]. CUR and N-CUR downregulated NF-κB, iNOS, TNF-α, Bax and caspase-3, and increased Bcl-2 and PCNA in the testes of Cu-administered rats. These findings demonstrated the anti-inflammatory and anti-apoptosis properties of CUR and pinpointed the superior activity of N-CUR in attenuating inflammation and apoptosis. CUR prevented testicular inflammation and apoptosis as reported in previous studies. For instance, CUR prevented apoptosis of testicular Leydig cells challenged with palmitic acid [74], and attenuated inflammation in the testes of methotrexate-intoxicated rats [75]. Similar to its antioxidant effect, the anti-inflammatory activity of CUR could be linked to the upregulation of Nrf2/HO-1 signaling. This notion is supported by the study of Boyanapalli et al. who reported that the anti-inflammatory activity of CUR was abolished in Nrf2^–/–^ mice and macrophages [76].

N-CUR showed a significant modulatory effect on LH, genes of steroidogenesis, AR, LPO, cellular antioxidants, Nrf2/HO-1 signaling, and inflammatory, proliferation and apoptosis markers when compared with CUR. This superior activity could be directly attributed to the improvement of its properties upon nanoformulation. When compared with the native form, N-CUR exhibited stronger antioxidant and anti-LPO efficacies than CUR in hepatoma cells lines [77], and attenuated inflammatory response in LPS-treated macrophages [78].

## 5. Conclusions

These results introduce compelling evidence for the deleterious effect of CuSO_4_ on testicular tissue, pituitary-gonadal axis, and steroidogenesis and the protective efficacy of CUR and N-CUR. Treatment with N-CUR and the native form prevented Cu-induced testicular tissue damage, oxidative stress, inflammation, and apoptotic cell death, and ameliorated cell proliferation, circulating levels of sex hormones, and steroidogenesis in rats. These effects were associated with activation of Nrf2/HO-1 signaling and enhanced antioxidants. N-CUR showed stronger protection when compared with CUR, an effect that is ascribed to the improved properties. Therefore, N-CUR is a potent protective agent against testicular toxicity caused by CuSO_4_, pending further investigations to explore other mechanisms.

## Figures and Tables

**Table 1:** Primers used for qRT-PCR.

Gene	GenBank Accession Number	Primers (5′-3′)	Amplicon Size (bp)
StAR	NM_031558.3	F: CCCAGATAGAGTTCGCCAGC R: TGGTGGGCAGTCCTTAACAC	84
CYP17A1	XM_006231434.3	F: TCAGTGACTGTGACCTGGGA R: GTGGAGCGGAGCAACTTCAA	122
3β-HSD	NM_001007719.3	F: TGTGCCAGCCTTCATCTAC R: CTTCTCGGCCATCCTTTT	145
17β-HSD	NM_054007.1	F: GACCGCCGATGAGTTTGT R: TTTGGGTGGTGCTGCTGT	140
NF-κB p65	NM_199267.2	F: CCTCATCTTTCCCTCAGAGCC R: GGTCCCGTGTAGCCATTGAT	189
iNOS	NM_012611.3	F: ATTCCCAGCCCAACAACACA R: GCAGCTTGTCCAGGGATTCT	112
TNF-α	NM_012675.3	F: AAATGGGCTCCCTCTCATCAGTTC R: TCTGCTTGGTGGTTTGCTACGAC	111
Caspase-3	NM_012922.2	F: GGAGCTTGGAACGCGAAGAA R: ACACAAGCCCATTTCAGGGT	169
BAX	NM_017059.2	F: AGGACGCATCCACCAAGAAG R: CAGTTGAAGTTGCCGTCTGC	166
BCL-2	NM_016993.1	F: ACTCTTCAGGGATGGGGTGA R: TGACATCTCCCTGTTGACGC	94
β-actin	NM_031144.3	F: AGGAGTACGATGAGTCCGGC R: CGCAGCTCAGTAACAGTCCG	71

## Data Availability

Data analyzed or generated during this study are included in this manuscript.
